# Rho signaling research: history, current status and future directions

**DOI:** 10.1002/1873-3468.13087

**Published:** 2018-05-24

**Authors:** Shuh Narumiya, Dean Thumkeo

**Affiliations:** ^1^ Department of Drug Discovery Medicine Medical Innovation Center Kyoto University Graduate School of Medicine Japan

**Keywords:** actin, actomyosin, C3 exoenzyme, myosin, Rho, ROCK, SRF, Y‐27632

## Abstract

One of the main research areas in biology from the mid‐1980s through the 1990s was the elucidation of signaling pathways governing cell responses. These studies brought, among other molecules, the small GTPase Rho to the epicenter. Rho signaling research has since expanded to all areas of biology and medicine. Here, we describe how Rho emerged as a key molecule governing cell morphogenesis and movement, how it was linked to actin reorganization, and how the study of Rho signaling has expanded from cultured cells to whole biological systems. We then give an overview of the current research status of Rho signaling in development, brain, cardiovascular system, immunity and cancer, and discuss the future directions of Rho signaling research, with emphasis on one Rho effector, ROCK*.

*The Rho GTPase family. Rho family GTPases have now expanded to contain 20 members. Amino acid sequences of 20 Rho GTPases found in human were aligned and the phylogenetic tree was generated by ClustalW2 software (EMBL‐EBI) based on NJ algorithm. The subfamilies of the Rho GTPases are highlighted by the circle and labeled on the right side. Rho cited in this review refers to the original members of Rho subfamily, RhoA, RhoB and RhoC, that are C3 substrates, and, unless specified, not to other members of the Rho subfamily such as Rac, Cdc42, and Rnd.

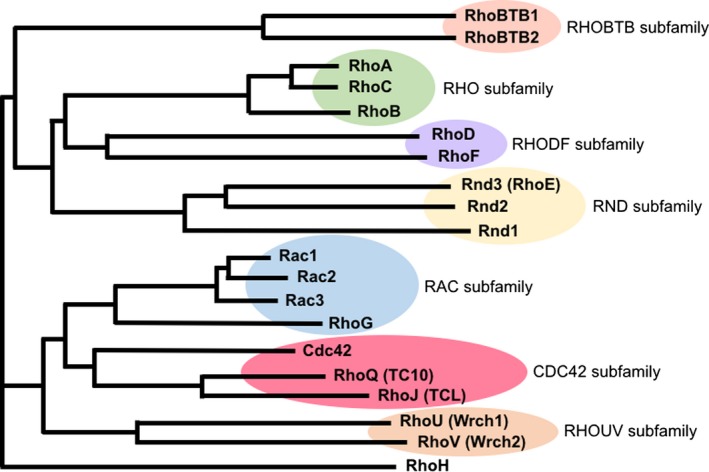

*The Rho GTPase family. Rho family GTPases have now expanded to contain 20 members. Amino acid sequences of 20 Rho GTPases found in human were aligned and the phylogenetic tree was generated by ClustalW2 software (EMBL‐EBI) based on NJ algorithm. The subfamilies of the Rho GTPases are highlighted by the circle and labeled on the right side. Rho cited in this review refers to the original members of Rho subfamily, RhoA, RhoB and RhoC, that are C3 substrates, and, unless specified, not to other members of the Rho subfamily such as Rac, Cdc42, and Rnd.

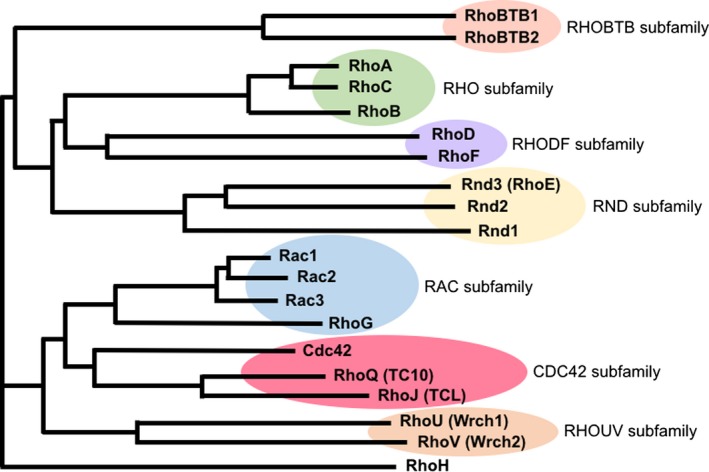

## Abbreviations

**ECM**, extracellular matrix

**LPA**, lysophosphatidic acid

**MLC**, myosin light chain

**MRTF**, myocardin‐related transcription factor

**PBMCs**, peripheral blood mononuclear cells

**ROCK**, Rho‐associated coiled‐coil containing kinase

**SRF**, serum response factor

## Discovery of Rho and botulinum C3 exoenzyme; the dawn of Rho research

In the mid‐1980s, there was a transition in signal transduction research. While prototypes of transmembrane signaling such as heterotrimeric G proteins and G‐protein coupled receptors, receptor tyrosine kinases, channels and transporters, had been identified and cloned, little was known on how the signals transmitted within the cell evoke cell responses such as proliferation, exocytosis and adhesion. Although involvement of GTP in some of these responses was reported, molecules bearing such GTP‐dependent functions were far from identification. An exception was Ras, which had been identified as retrovirus‐coded oncogenes encoding a 21 kDa GTP‐binding protein, but its action mechanism also remained an enigma. In this situation, Madaule and Axel serendipitously identified the first Ras homolog in Aplysia, and named it Rho 1. They further detected Rho genes in human and rat, and suggested that those of human possibly consist of three members, which were later named RhoA, B, and C. However, without any biological findings, little attention was paid to it.

One approach to discover mechanisms and principles working in biological processes is to find out pharmacological tools interfering with such processes by specifically targeting the molecules involved. Given the demonstrated usefulness of cholera toxin and pertussis toxin as probes of heterotrimeric G‐proteins, Gs and Gi/Go, respectively 2 and the fact that most of the bacterial toxin ADP‐ribosyltransferases target GTP‐binding proteins 2, we sought a novel bacterial ADP‐ribosyltransferase activity, and identified in preparations of botulinum C1 and D toxin an enzyme that ADP‐ribosylates a 22 kDa GTP‐binding protein in mammalian cells. We reported these findings in the *Journal of Biological Chemistry* on February 5, 1987 3. To our surprise, the same enzyme activity was reported in the February 9, 1987, issue of *FEBS Letters* by Aktories and collaborators 4. They later further reported that this ADP‐ribosyltransferase is distinct from botulinum neurotoxins and named it C3 exoenzyme 5. Almost 1 year later, Rubin *et al*. confirmed these findings and additionally reported that the C3 treatment induced morphological changes in cultured cells, typically the rounding up of fibroblasts and epithelial cells and the neurite extension of neuronal cells 6, which we also noted separately 7, 8. However, the identity of the target GTP‐binding protein was not elucidated until we purified and identified it as Rho 9, 10, 11. Our studies thus merged researches on Rho and C3. Chardin *et al*. then reported that actin microfilaments were lost in C3‐treated round‐up Vero cells 12. The next question was therefore how the morphological phenotype of C3‐treated cells is related to the function of Rho and how Rho is involved in actin microfilament assembly. Because little was known about the difference between the Rho isoforms, most of the early studies in the field were focused on RhoA.

## Rho as a molecular switch for actin cytoskeleton reorganization

According to the analogy to Ras, it was thought that Rho is activated from the GDP‐bound form to the GTP‐bound form to exert its actions. So, the above question could be addressed by comparing the C3 phenotype with that induced by active Rho. This was exactly where Alan Hall and collaborators entered the field, using their expertise in Ras biochemistry and microinjection technique. They microinjected Val^14^‐RhoA, a presumably active RhoA mutant with reduced GTPase activity, and found extensive actin filament assembly in the contracted body of microinjected cells 13. Although this Val^14^‐RhoA phenotype appeared opposite to the above phenotype of C3‐treated cells, it was not clear at this time what kind of cell structures and what kind of cell response they represent. Ridley and Hall addressed these points 14. They showed that the actin filament structure induced by active RhoA in fibroblasts represents actin stress fibers linked to focal adhesions, that these structures are induced by the addition of serum to the cells, and that this induction was inhibited by microinjection of C3 or ADP‐ribosylated Rho, thus making clear that Rho works as a molecular switch in stimulus‐induced stress fiber formation. By this time, homology cloning and protein purification identified a variety of Ras‐related GTP‐binding proteins in mammals and yeast. Didsbury *et al*. 15 isolated two cDNAs highly homologous to Rho from HL‐60 kibrary, and erroneously named Rac (Ras‐related C3 substrate) 1 and 2. Ridley and Hall therefore extended their study to examine the function of Rac1, and reported that Rac1 induces a different actin filament structure, membrane ruffles, in response to PDGF 16. Their works thus established the paradigm that Rho GTPases function as molecular switches for actin reorganization.

Focal adhesion induced by Rho is the multi‐protein complex of integrin and associated proteins, which is clustered by the force of actomyosin bundles ligated to this complex, and serves as adhesion to extracellular matrix (ECM). Therefore, Rho‐induced focal adhesion formation facilitates cell adhesion to ECM by increasing the integrin avidity. We confirmed RhoA action with platelet aggregation as an example, which is mediated by binding of platelet integrin GPIIb/IIIa to soluble ECM ligands such as fibrinogen 17.

Other notable examples of Rho‐regulated cell processes were neurite retraction and cytokinesis. In the above work on stress fibers, Ridley and Hall identified a major RhoA activating factor in serum as lysophosphatidic acid (LPA). The biological activity of this lipid was found by Wouter Moolenaar, who also found that the addition of LPA induces neurite retraction in neuroblastoma cells. Hearing his talk at our Department seminar, we noticed that this LPA phenotype was opposite to neurite extension by the C3 treatment. We therefore collaborated and found that RhoA mediates neurite retraction induced by LPA 18. We also collaborated with Issei Mabuchi on the role of Rho in cytokinesis, and found that the C3 treatment aborted cytokinesis by abolishing the contractile ring, indicating that Rho links nuclear division to cytoplasmic division through induction of the contractile ring 19. Kishi *et al*. analyzed the division of Xenopus embryos and reached the same conclusion 20.

Intriguingly, Treisman and collaborators found that LPA also activates the transcription factor “serum response factor (SRF)” and this activation also requires functional RhoA 21. Their later studies showed that SRF activity is regulated by SRF transcriptional coactivator myocardin‐related transcription factor (MAL/MRTF) and that the interaction between SRF and MAL/MRTF is inhibited by the binding of MAL/MRTF to G‐actin 22. Upon RhoA activation, G‐actin is incorporated into F‐actin and MAL/MRTF is subsequently released from G‐actin. This facilitates the formation of SRF‐MAL/MRTF complexes and thus the activation of SRF‐dependent transcription of genes that are involved in a variety of cellular processes such as cell migration, cell proliferation and cell differentiation 23.

## Search and identification of Rho effectors; elucidation of molecular mechanisms of Rho actions

**Figure 1:**
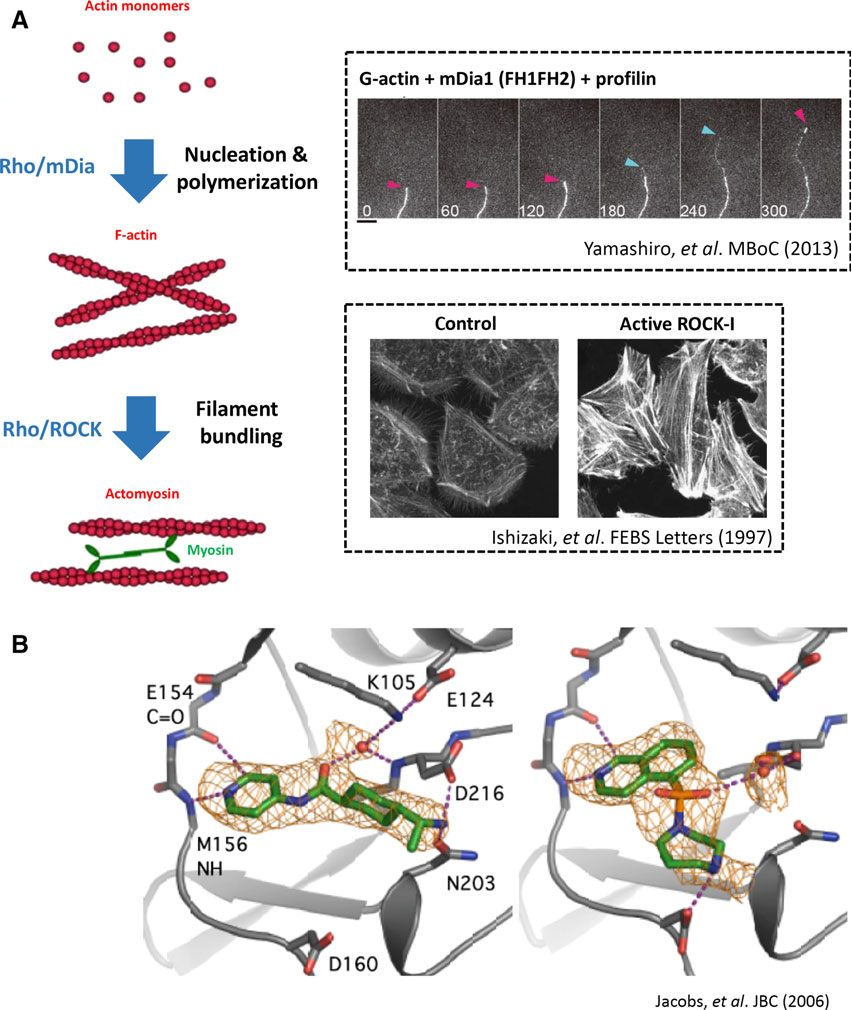
(A) Simplified scheme depicting the actions of mDia and ROCK in Rho‐mediated actin remodeling. Upon the activation by Rho, mDia promotes actin nucleation and polymerization to form actin filaments and ROCK activates myosin to bundles actin filaments. The upper right box shows the FH1FH2 of mDia1‐catalyzed actin polymerization *in vitro*. Red arrowheads indicate the mDia1‐free barbed end of F‐actin growing at slow rate, and blue arrowheads indicate the barbed end undergoing mDia1 (FH1FH2)‐dependent fast growth. Times are indicated in seconds. Scale bar, 5 μm. Modified from *Yamashiro S, et al*. MBoC 25, 1010–1024 (2014). The lower right box shows F‐actin staining of HeLa cells overexpressing vector control or active ROCK‐I. Note that F‐actin bundles are extensively induced in active ROCK‐I overexpressed cells. Modified from *Ishizaki T, et al*. FEBS Lett. 404, 118–124 (1997). (B) Crystal structures of Y‐27632‐bound (left) and fasudil‐bound (right) kinase domain of ROCK‐I. Modified from *Jacobs M, et al*. JBC 281, 260–268 (2006).

Both stress fibers and the contractile ring are composed of actomyosin bundles and neurite retraction is caused by their contraction. These findings led us to hypothesize that the action of Rho is to make actin filaments from actin monomers and then cross‐link them by activating myosin for contraction. So, the next issue of Rho research was to find out molecules and mechanisms underlying these steps, which are presumably carried out by effector molecules downstream of Rho. Since the GTP‐bound and not GDP‐bound Rho exerts its actions, effector molecules were presumed to bind selectively to the GTP‐bound Rho. Isolation of small GTPase effectors by such selective binding was heralded by Louis Lim and collaborators, who isolated p65PAK (PAK1) as a Cdc42/Rac effector in 1994 24. Using selective binding to the GTP‐bound Rho in ligand overlay assay or yeast two hybrid systems, we isolated several Rho effectors 25, 26, 27, 28, 29. One of them is Rho‐associated coiled‐coil containing kinase (ROCK), which consists of two isoforms, ROCK‐I (ROCK1) and ROCK‐II (ROCK2) 26, 30. The same enzymes were isolated and called ROK and Rho‐kinase by Louis Lim's group 31 and Kozo Kaibuchi's group 32, which correspond to ROCK‐I and ROCK‐II, respectively. Another effector we isolated was a mammalian homolog of *Diaphanous* (mDia) 29 *,* which belongs to the formin family and consists of three isoforms 33. Expression of active ROCK produced actomyosin bundles reminiscent of stress fibers and extensive formation of focal adhesions in HeLa cells 34, 35 presumably through activation of myosin (see below), and expression of active mDia increased the density of actin filaments 29, suggesting that it induces actin polymerization. The actin nucleation/polymerization activity of the formin family was later shown in yeast formin, Bni1p 36 and then mDia 37. Co‐expression of ROCK and mDia produced beautifully aligned stress fibers in HeLa cells, thus reproducing the action of Rho on stress fiber formation 38. Thus, mDia and ROCK are thought as main effectors in Rho‐induced actin reorganization (Fig. 1).

## Discovery of Y‐27632, a specific ROCK inhibitor; a pivotal point from cell biology to physiology

Smooth muscle contraction is triggered by myosin light chain (MLC) phosphorylation. The involvement and mechanism of ROCK in myosin activation was revealed both biochemically and pharmacologically by the analysis of the so called “calcium‐sensitization pathway” of smooth muscle contraction. This pathway augments contraction at a fixed intracellular calcium ion concentration [Ca^2+^]_I_, and was previously demonstrated to involve GTP, RhoA and myosin phosphatase 39, 40. Kaibuchi's group showed that Rho kinase/ROCK phosphorylates myosin‐binding subunit of myosin phosphatase, thus inactivating the phosphatase and consequently raising MLC phosphorylation and contraction 41. The involvement of ROCK in this process was also confirmed pharmacologically using Y‐27632. Y‐27632 was developed as a compound that inhibits the calcium‐sensitization pathway of smooth muscle contraction, and the photo‐affinity cross‐linking and the assay on recombinant ROCK identified it as a selective ROCK inhibitor 42. This inhibitor not only inhibited the calcium‐sensitization of smooth muscle selectively but also abolished RhoA‐induced formation of stress fibers and focal adhesions. This compound further inhibits the RhoA‐mediated neurite retraction in neuroblastoma cells and analysis of this effect revealed the RhoA‐mediated inhibition of actin depolymerization through the ROCK‐LIM kinase‐cofilin pathway 43. Thus, identification of Rho effectors and a ROCK‐specific inhibitor facilitated elucidation of molecular mechanisms of Rho‐mediated cellular responses. The impact of the discovery of Y‐27632, however, was not limited to analysis of cultured cells but also on intact animals, in which Y‐27632 is used to examine possible involvement of Rho‐ROCK signaling in various physiological and pathophysiological processes as described below. Thus, the discovery of Y‐27632 made a pivotal point in Rho research.

## Rho signaling research in current status

With the spread of the knowledge on Rho signaling in various cell processes and the advent of various dissecting tools, that is, drugs, expression constructs, RNAi and transgenic, and knockout animals, Rho signaling research has spread widely to all areas of biology. Here, we select several fields and overview their current status.

### Development and regeneration

**Figure 2:**
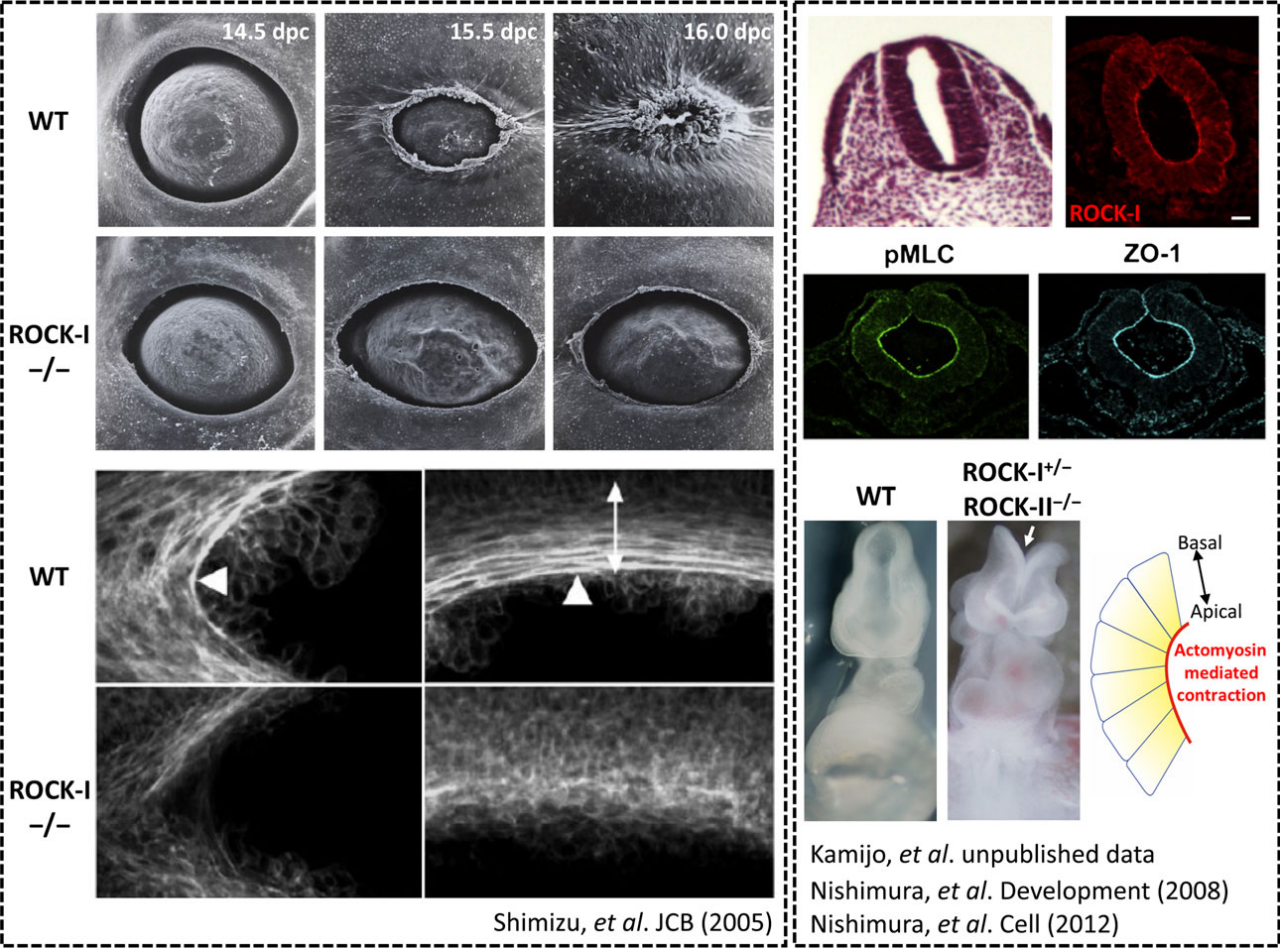
Examples of the ROCK actions in development. The left box shows the impaired eyelid closure phenotype of ROCK‐I^−/−^ embryos. Scanning electron micrographs of the eyes of the WT and ROCK‐I^−/−^ embryos are shown on the top and whole‐mount F‐actin staining of the eyelids of WT and ROCK‐I^−/−^ embryos are shown on the bottom. Note that F‐actin bundles encircling the eye (arrowheads) are impaired in ROCK‐I^−/−^ embryos. Modified from *Shimizu Y, et al*. JCB 168, 941–953 (2005). The right box shows the role of ROCK in neural tube closure. H&E staining of mouse embryo neural tube is shown on the top left (H. Kamijo, T. Ishizaki, D. Thumkeo, S. Narumiya, *et al*., unpublished results). The upper three panels show immunofluorescence micrographs of chick embryo neural tube during neural tube closure. Modified from *Nishimura T, et al*. Development 141, 1987–1998 (2008) and *Nishimura T, et al*. Cell 149, 1084–1097 (2012). Note concentration of ROCK‐I and pMLC on the apical surface as marked by ZO‐1 staining. The lower panels show stereomicroscope micrographs of 9.5 dpc mouse embryo neural tube (H. Kamijo, T. Ishizaki, D. Thumkeo, S. Narumiya, *et al*., unpublished results). Note impaired neural tube closure of ROCK‐I^+/−^; ROCK‐II^−/−^ mouse embryo (white arrow). A model proposes the role of ROCK‐mediated actomyosin on the apical surface of neuroepithelium during neural tube closure is shown on the bottom right.

Rho signaling functions critically in several developmental processes (Fig. 2). Its typical mode of action here is to form actomyosin bundles traversed intercellularly by connecting neighboring epithelial cells through cell‐cell adhesion and contract them for tissue morphogenesis and maintenance. Both ROCK and mDia are involved. For example, mice‐deficient in either ROCK‐I or ROCK‐II fail in closure of the eyelid and the ventral body wall and are born with the ‘eyes open at birth’ and omphalocele phenotype. In these mice, actin cables that encircle the eye in the epithelial cells of the eyelid as well as those encircling the umbilical ring are disorganized 44, 45, 46. Although each of ROCK‐I and ROCK‐II exhibits distinct roles in many circumstances described below, they apparently act functionally redundant in these closure processes. In mDia1/3 double KO mice, apical actin belts in neuroepithelial cells were attenuated and their apical adherens junctions were impaired, resulting in the loss of apical‐basal polarity of neuroepithelial cells and periventricular hyperplasia 47. When such apical actomyosin cables contract, it causes apical constriction of epithelial cells and, making cuboidal cells to trapezoid, bending tissues and forming three‐dimensional structures such as tube and invagination. This is seen in gastrulation and neurulation, where Rho, ROCK and mDia function 48. In chick neural tube formation, cadherin, Celsr1, recruits PDZ‐RhoGEF at the mediolateral adherens junctions to upregulate Rho‐ROCK signaling and cause actomyosin contraction apically in a planar‐polarized manner 49, 50. A similar role of Rho‐ROCK signaling in planar cell polarity was reported in Drosophila germ band extension 51. Furthermore, ROCK functions also in making convex invagination in optic‐cup‐like structure formation from ES cells in culture 52. In addition, the polarized localization of ROCK causes biased actomyosin activity in a single cell, and this mechanism operates crucially in asymmetric cell division of Drosophila neural stem cells 53.

YAP and TAZ are effectors of the Hippo signaling involved in control of organ size, stem cell renewal, regeneration and cancer 54. They are also involved in mechanotransduction, and translocate to the nucleus on sensing of ECM stiffness and cell spreading in Rho‐ and actomyosin tension‐dependent manners 55. Requirement of Rho signaling is also observed in activation of YAP/TAZ by Wnt signaling, in which Wnt activates Rho through FZD‐ROR‐Gα12/13 pathway and inhibits Lats1/2 56. This Rho‐mediated activation of YAP/TAZ is required for long‐term survival and expansion of human ES cells cultured *en bloc* 57. Interestingly and paradoxically, dissociated human ES cells exhibit Rho‐ROCK‐mediated hyperactivation of myosin and the resultant contraction induces their death, which can be rescued by Y‐27632 58, 59, 60.

### Brain morphogenesis and function

Rho signaling is involved in brain morphogenesis and functions including axonogenesis, neuronal migration and synaptic plasticity. Neurite retraction in cultured neuron is mediated by ROCK 61, 62 and ROCK2 KO mice exhibited enhanced axonogenesis after spinal cord injury and recovered faster than the control mice 63. The Rho‐ROCK signaling is now recognized as the final common pathway to limit axonogenesis in CNS trauma and the potential of ROCK inhibitor in axonal regeneration is being examined 64. Notably, this Rho‐ROCK action operates in not only such pathophysiological process but also neuronal development. Kaibuchi's group recently showed that the growing axon transmits long‐range Ca^2+^ waves to other neurites, activates RhoA‐ROCK pathway there and suppresses their axonogenesis to allow the formation of a single axon in neurons 65. Rho signaling also works in neuronal migration; mice deficient in mDia1 and 3 in combination exhibit deficit in tangential migration of interneuron precursors from subventricular zone to the olfactory bulb 66. Moreover, Rho signaling plays critical roles in synaptic plasticity. The dendritic spine is the site of memory. Rac and Rho work antagonistically in shaping dendritic spines, growth and shrinkage, respectively, and ROCK mediates the latter Rho action 67. By this action, ROCK apparently is involved in some types of mental retardation, where a Rho GTPase activating protein named oligophrenin‐1 is mutated; down‐regulation of oligophrenin‐1 results in spine shrinkage, which is rescued by ROCK inhibitor 68. In addition to these postsynaptic actions, ROCK functions in synaptic vesicle retrieval in the presynaptic terminal to contribute to the homeostatic balance of vesicle exocytosis and endocytosis at synapse 69. Furthermore, Rho signaling is involved in plasticity of the presynaptic terminal. Deguchi *et al*. 70 found that social isolation of mice induces inactivation of *Nucleus accumbens* neurons, which then leads to mDia and ROCK‐dependent contraction of their terminals in the *Ventral tegmental area* and reduces synaptic transmission there, which causes enhanced anxiety behavior in these animals. These actions of Rho‐ROCK signaling could be involved in synaptic plasticity in the lateral amygdala associated with fear conditioning as well as that in prelimbic prefrontal cortex associated with goal‐directed decision making, both of which is sensitive to ROCK inhibition 71, 72.

### Cardiovascular system

Since Y‐27632 was discovered through screening for compounds that inhibit calcium sensitization of arterial contraction and shown to lower blood pressure in various rat models of hypertension 42, much interest has arisen naturally in the role of Rho‐ROCK signaling in the cardiovascular system. Such interest was boosted further by the finding that fasudil, a drug approved in Japan for treatment of vasospasm after subarachnoid hemorrhage, is also a ROCK inhibitor 73, which has stimulated studies not only in animals but also in humans. For example, fasudil is effective in decreasing the frequency of attacks in stable angina patients 74 and attenuating coronary artery vasospasm in patients with vasospastic angina, the effect reproduced in the porcine model with enhanced myosin phosphatase activation 75. These findings led to the proposal of a role of ROCK in coronary vasospasm. In idiopathic pulmonary hypertension, ROCK2 is highly expressed in pulmonary arteries of patients and fasudil treatment induces acute pulmonary vasodilation 76. Consistently, the selective loss of ROCK2 in vascular smooth muscle prevents development of chronic hypoxia‐induced pulmonary hypertension in mice, suggesting the causative relation of ROCK2 in this disease. Other studies using ROCK1 and ROCK2 KO mice and ROCK inhibitors in various animal models indicate the involvement of ROCK in diabetic vasculopathy, ischemia/reperfusion injury, heart failure, cardiac hypertrophy, and fibrosis 77. Interestingly, in aortic constriction model, the cardiomyocyte‐specific deletion of ROCK2 suppressed the cardiac hypertrophy 78, while the ROCK1 haploinsufficiency did not prevent cardiac hypertrophy but reduced fibrosis under the same condition 79. A profibrotic role of ROCK is also reported in various organs including lung 80.

### Immunity

Early studies on the role of Rho in the immune system focused on its role in development, activation and migration of T cells and B cells 81. For example, studies on mice expressing C3 or active RhoA in T cell lineages suggested that Rho is important in thymocyte expansion but dispensable for T cell development itself 82, 83. Indeed, deficiency of RhoA in T cells does not completely suppress but significantly attenuates in T cell receptor‐dependent proliferative response 84. Inactivation of Rho and treatment with ROCK inhibitors impairs T cell migration. The most notable feature is deficit in transendothelial migration due to impaired uropod retraction 85, a feature shared with neural precursors, macrophages, neutrophils and cancer cells 66, 86, 87, 88. Recent studies have focused more on the role of ROCK in the differentiation of T helper cell (Th) subsets and its pathological significance 89. Rho‐ROCK signaling appears to function in both the sensitization phase and effector phase of Th2‐dependent allergic inflammation. T cell‐specific deletion of RhoA impaired Th2 differentiation but not Th1 differentiation *in vitro* and prevented OVA‐induced allergic inflammation *in vivo* with reduction in IgE level, cell infiltration in the airway and Th2 cytokine production 84. Administration of fasudil all through the experimental period mimicked these effects of RhoA deficiency. In addition, inhalation of Y‐27632 during the allergen challenge could suppress airway constriction and hypersensitivity and partially prevented cell infiltration to the airway in an OVA‐induced asthma model of guinea pig 90. These effects of Y‐27632 may well be due to the suppression of enhanced calcium sensitization of airway smooth muscle contraction induced by allergen‐sensitization 91, and effects of ROCK inhibition on chemotaxis of inflammatory cells. Studies using ROCK hetero‐deficient mice indicate that both ROCK1 and ROCK2 in lymphocytes and nonlymphocyte cells are involved in these processes 92, 93.

The more intriguing findings on the role of ROCK in Th subsets are that of ROCK2 in Th17 cells. This was first reported by the Pernis group, who found that ROCK2 is selectively activated in CD4^+^ T cells under Th17 skewing conditions and phosphorylates interferon regulatory factor‐4 (IRF‐4) to facilitate Th17 cell differentiation and that administration of ROCK inhibitor suppresses production of Th17 cytokines, IL‐21 and IL‐17, and ameliorates symptoms in autoimmune model mice 94. Concurrently, Kadmon Pharmaceuticals ran Phase 1 clinical trial of a ROCK2‐selective inhibitor, KD025 95, 96. Zanin‐Zhorov *et al*. 96 analyzed responses of peripheral blood mononuclear cells (PBMCs) from human subjects in the above trial and found that KD025 administration *in vivo* significantly inhibited *ex vivo* secretion of IL‐21 and IL‐17 from activated PBMCs. They confirmed this effect in human CD4^+^ T cells stimulated *in vitro* under the Th17 skewing conditions, and found that it is through suppression of STAT3 phosphorylation. Intriguingly, while KD025 suppresses Th17 differentiation through decreased STAT3 phosphorylation, it accelerates regulatory T cell (Treg) differentiation through enhanced STAT5 phosphorylation. The effect of KD025 to suppress IL‐17 and IL‐21 production was then confirmed in PBMCs from patients with rheumatoid arthritis, graft‐versus‐host disease, systemic lupus erythematosus and inflammatory bowel diseases 96, 97, 98, 99. Further, recent Phase 2 studies showed that oral administration of KD025 reduces clinical scores in psoriasis patients with concomitant decrease in plasma levels of IL‐17 and IL‐23 and increase in that of IL‐10 100.

### Cancer

Since many *dbl*‐containing Rho GEFs were isolated by transformation assay of cultured fibroblasts 101 and Rho‐GAP domain‐containing DLC‐1 (Deleted in Liver Cancer‐1) is downregulated in various tumors and is regarded as a tumor suppressor 102, Rho GTPases have been implicated in cell transformation and oncogenesis. Indeed, earlier works showed requirement of Rho GTPases in Ras‐mediated cell‐transformation 103. Notably, while these studies used GTPase‐deficient G14V or Q63L in RhoA and G12V or Q61L Rac1 analogous to oncogenic Ras mutations, such mutations in Rho GTPases have not been detected in clinical cancer. On the contrary, fast cycling P29S, P29L, and P29Q mutations in Rac1 have been identified by high‐throughput sequencing of clinical human cancers 104, 105, 106, the G17V RhoA mutation frequent found in T cell lymphomas 107, 108 and the Y42C RhoA mutation recurrently in diffuse gastric cancer 109. Since biochemical properties of these RhoA mutation are not clear, how these RhoA mutations induces oncogenesis is an interesting question to be solved 110. In addition to these mutations in Rho GTPases, more than 600 somatic coding mutations in ROCK1 and ROCK2 have been identified in human cancers and downregulation of miRNAs targeting ROCK1 and ROCK2, and, consequently upregulation of ROCKs has been shown in malignant tissues 111. This enhanced ROCK signaling could facilitate cell transformation for tumor cell survival and growth. Y‐27632 treatment was reported to inhibit transformation of NIH3T3 cells by Dbl and Ras 112, and conditional deletion of ROCK1 and ROCK2 in combination was shown to inhibit transformation of cells derived from Ras‐driven lung tumors and Raf‐driven melanomas 113. ROCK signaling likely functions in tumor cell invasion and metastasis, which was first shown in the peritoneal tumor dissemination model 88. Tumor cells can migrate as single cells or collectively as a cluster. Experiments in three‐dimensional matrix suggest that tumor cells adopt two different modes of single‐cell migratory mechanisms, Rac‐mediated elongated mesenchymal migration and ROCK‐mediated rounded amoeboid migration, which are interconvertible and utilized in the context‐dependent manner 114. ROCK signaling also enables tail retraction in transendothelial and transepithelial migration of tumor cells 88. ROCK can also remodel extracellular matrix in tumor microenvironment for tumor invasion. Sanz‐Moreno *et al*. 115 showed in collagen matrix model that increased ROCK signaling induced by cytokine contracts stromal fibroblasts to create tracks for collective migration of squamous carcinoma cells. Rath *et al*. 116 showed that ROCK activation in mouse pancreatic ductal adenocarcinoma cells increased their invasive growth into a three‐dimensional collagen matrix by extensive induction of matrix metalloproteinases and increased matrix remodeling. These findings combined together suggest that Rho‐ROCK signaling is not the primary cause but critical for several important phenotypes of cancer, which may be exploited therapeutically in combination with other anticancer therapies 111.

## Future prospects

**Figure 3:**
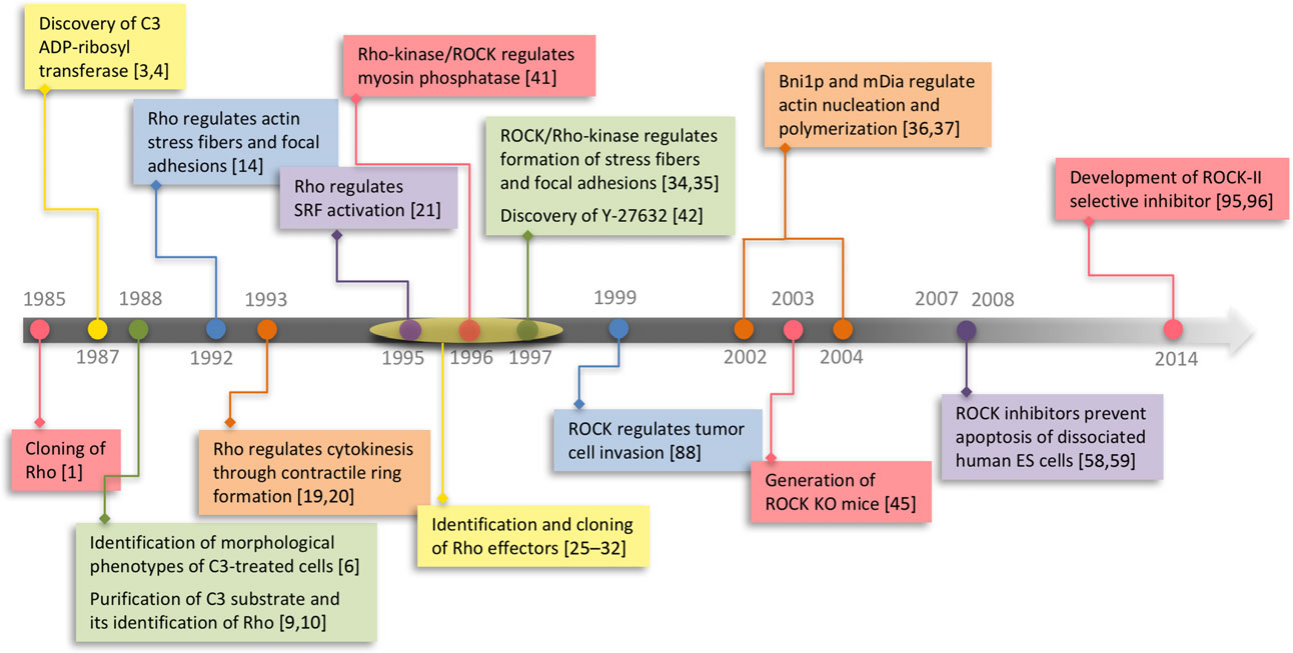
Milestone discoveries in Rho signaling research.

Although a substantial amount of work has been done since the discovery of Rho more than 30 years ago, elucidating important physiological roles of this GTPase family and their action mechanisms as described above (Fig. 3), the whole picture of the biology of Rho, how each member of the Rho family GTPases is activated under what conditions in which tissue, exerts which body function by acting on which effectors, is still far from complete. Generation and analysis of tissue‐specific conditional knockout mice deficient in each member of Rho GTPases, effectors and regulatory proteins, GEFs and GAPs, could help our understanding. This is true even for each of classic Rho member, RhoA, RhoB, and RhoC. Most of the earlier studies on Rho described above have been carried out on RhoA, and it is not certain whether other Rho members exert the same actions in the cell. Although some studies suggest the redundant roles between these RhoA, RhoB, and RhoC isoforms 117, 118, their different cellular localization and different regulatory modes of expression strongly suggest that they play also context‐ and localization‐dependent distinct roles in the cell and possibly in the body 119. For example, although these three Rho members similarly interact with Rho effectors thus far identified, RhoA, RhoB, and RhoC act on different effectors, namely ROCKi/2, integrins and formin FMNL3, respectively, and exert different functions in cancer cell migration and morphogenesis 120. RhoB shows unique endosome localization, and is suggested to exert different functions from RhoA and RhoC 121. Furthermore, while RhoA‐null mice are embryonic lethal 122, RhoB‐null and RhoC‐null mice are viable 123, 124. There are also issues on atypical Rho GTPases such as Rnd proteins, RhoBTB proteins, RhoH, RhoU and RhoV 119. More remains to be clarified on their regulatory and effector mechanisms, and again their body function. As for Rho GTPase activation, there are 70 Rho GEFs of Dbl homology and 10 DOCK homologs. Studies have been in progress elucidating what physiological context each GEF is activated and contributes to, one classic examples being p115 RhoA‐GEF coupling to Gα12/13 for cell contraction 125. Given such importance of Rho and Rho regulators and effectors in many biological processes, the studies on the dynamics of their activation and termination, that is how Rho and Rho regulators and effectors are activated at the right time and place, and how this signal propagates in the living cell in a variety of physiological processes, is an important issue. The challenge is nanoscale imaging of spatiotemporal actions of Rho‐Rho effectors not only in living cultured cells but also hopefully in intact tissues or intact body. Such studies combined with the systematic analysis on the functions of Rho signaling at respective sites will help to elucidate the pictures of Rho actions in the body. Finally, our understanding on the roles of Rho and Rho effectors in pathophysiology has just begun. As illustrated by the recent discovery of the role of ROCK2 in autoimmunity and the therapeutic effects of its selective inhibitor 100, unraveling the roles of Rho and Rho effectors further in various pathophysiological settings by the above approach may reveal unappreciated therapeutic possibilities.

## Acknowledgements

This work was supported in part by Grants‐in‐aid for Scientific Research from the Ministry of Education, Culture, Sports, Science and Technology of Japan.
